# Editorial: Glia in Health and Disease

**DOI:** 10.3389/fnmol.2019.00063

**Published:** 2019-03-19

**Authors:** Margaret S. Ho, Alexei Verkhratsky, Shumin Duan, Vladimir Parpura

**Affiliations:** ^1^School of Life Science and Technology, ShanghaiTech University, Shanghai, China; ^2^Faculty of Biology, Medicine and Health, The University of Manchester, Manchester, United Kingdom; ^3^Center for Basic and Translational Neuroscience, Faculty of Health and Medical Sciences, University of Copenhagen, Copenhagen, Denmark; ^4^Achucarro Center for Neuroscience, IKERBASQUE, Basque Foundation for Science, Bilbao, Spain; ^5^Department of Neurobiology, School of Medicine, Zhejiang University, Hangzhou, China; ^6^Department of Neurobiology, The University of Alabama at Birmingham, Birmingham, AL, United States

**Keywords:** glia, astrocytes, ALS, OPC, microglia

Half a billion of years of evolution of the central nervous system (CNS) resulted in emergence of two highly integrated and interconnected cellular networks (Verkhratsky and Nedergaard, 2016) of neurones (executive arm of the CNS) and neuroglia (CNS homoeostatic and defensive division). Concerted activity of these networks underlies CNS function and output including behavior, intelligence, emotions and consciousness. Evolutionary specialization produced division of function in which neurones provide for information processing and creation of circuit-mediated behavior, whereas neuroglia assume full responsibility for homeostatic support of the nervous system. In addition, neuroglia provide (through conserved programmes of activation) for the defense of the nervous tissue. These distinct functions are reflected by distinct physiology of these two types of cells. Neuronal signaling relies on electrical excitability mediated by voltage-gated ionic channels and fast (millisecond range) synaptic transmission. Glia employ controlled fluctuations in intracellular ions and messengers; intercellular fluxes of these molecules underlie long-range signaling in glial syncytia that occur (within seconds) in the form of propagated ionic or metabolic (slow) waves (Verkhratsky and Nedergaard, 2018). Intercellular communications between the two networks are mediated by chemical transmission and a complement of receptors; neuroglial cells are capable to perceive neuronal activity and to secrete ions and signaling molecules [although secretion mechanisms/tool sets differ from that in synaptic terminals—(Verkhratsky et al., 2016)].

The homeostatic and defensive capacity of neuroglia stipulates their fundamental role in neuropathology. Conceptually, the glial component is present in every type of neurological disease, with glial contribution being either primary and/or secondary. Astrocytes undergo several types of pathological transformation from pathological remodeling and atrophy/degeneration with a loss of function to astrogliotic transformation (Verkhratsky et al., 2013; Sofroniew, 2014b; Pekny et al., 2016; Verkhratsky and Parpura, 2016); the latter producing numerous reactive phenotypes with neuroprotective or neurotoxic features (Pekny and Pekna, 2014; Sofroniew, 2014a; Liddelow et al., 2017). Microglial cells, that constantly scan brain parenchyma for damage or pathogen-associated molecular patterns, respond to lesions by highly complex activation that produces multiple neuroprotective or neurotoxic phenotypes (Kettenmann et al., 2011; Salter and Stevens, 2017). Finally, oligodendroglia and their precursors (also known as NG2 glia) respond to pathology with Wallerian degeneration, proliferation, and remyelination (Sun et al., 2010; Catenaccio et al., 2017).

This Research Topic aims to address the role of neuroglia in healthy and diseased nervous tissue through a collection of 28 articles that include 2 mini-reviews, 4 reviews, 1 opinion, 19 original research article, 1 Perspective, and 1 Protocol. These articles provide a comprehensive overview on a broad spectrum of molecular and cellular basis of glial function in normal and pathological CNS.

The Research Topic discusses some of the physiological functions of neuroglia. The opinion on Ca^2+^-dependent and Ca^2+^-independent release of ATP from murine astrocytes is discussed by Xiong et al. while several articles discuss the regulation and molecular mechanisms of glial plasticity (Boerboom et al.; Lalo et al.; Wang and Parpura). Glial metabolism and other aspects of murine glial function were also included. A new signaling pathway associated with extracellular lactate, lactate receptor, and subsequent increase in cyclic-AMP was found to modulate astroglial metabolism by enhancing aerobic glycolysis (Vardjan et al.). In Zebrafish (Zhang et al.), microglial cells were shown to mediate clearance of apoptotic endothelial cells associated with vessel pruning. A novel method to study micropinocytosis in fly, *Drosophila*, hemocytes, phagocytic cells similar to microglia in mammals has been also described (Chen et al.).

Both astrocytes and microglia contribute to acute pathology such as ischemic stroke and epilepsy. Astrocytic K_ir_4.1, an inwardly rectifying K^+^ channel, is central to regulation of K^+^ buffering to rebalance the blast of increased extracellular K^+^ concentration during ischemic conditions (Milton and Smith). In ischaemic conditions, microglia get activated by microRNA miR-145-5p/ Nuclear receptor related 1 protein (Nurr1)/Tumor necrosis Factor-α cascade which can be used to define a novel therapeutic strategy to relieve neuronal damage as a consequence of microglial activation (Xuemei et al.). In the rat lithium-pilocarpine model of status epilepticus (SE), a significant reduction in the number of distal astrocytic branches were detected upon SE induction, suggesting that astrocytic atrophy correlates with SE-induced epileptic pathological conditions (Plata et al.). During central post-stroke pain, the Stromal cell-derived factor (SDF)-1 (CXC chemokine ligand-12)—(CXC chemokine receptor CXCR4) signaling is up-regulated and provide positive feedback to regulate glial-glial and glial-neuronal interactions (Fei et al.). Astrocytes are also involved in chronic pathologies; in particular they can mediate noradrenergic pathways responsible for neurodegeneration and cognitive decline in Alzheimer's disease (Leanza et al.).

Microglia-mediated response of the CNS significantly contributes to various chronic pathologies, including neurodegeneration. Articles assembled in this Research Topic indicate that microglial activation (induced by exposure to lipopolysaccharide, LPS) can be ameliorated by the natural plant compounds polyphenols and the glucagon like peptide-1 receptor (GLP-1R) agonist exendin-4 (EX-4) upon LPS exposure (Gullo et al.). Similar effects were exerted by caffeine/modafinil in a rat model of sleep deprivation; the treatment led to a significant improvement of the anxious behavior (Wadhwa et al.). As for the identification of molecular components involved in microglia-mediated pathological processes, the Dendritic cell-derived factor 1 (Dcf1), a factor in neural stem cell differentiation, glioma apoptosis, and dendritic spine formation, microglial lectins including galectins, siglecs, mannose-binding lectins (MBLs) as well as other glycan binding proteins, all play pivotal role in regulating microglia contribution to pathology (Siew and Chern; Wang et al.).

Glia contribute to many diseases. For instance, methyl CpG binding protein 2 (MeCP2) deficiency in various types of glia causes various defects and anomalies that contribute to Rett syndrome (Xu-Rui et al.). Activation of P2X_7_ purinoreceptor triggers microglial response through autophagy and mammalian target of rapamycin (mTOR) pathway in amyotrophic lateral sclerosis (ALS) mouse model with mutated (G93A) superoxide dismutase; this signaling cascade could represent a potential target for modulating ALS progression (Fabbrizio et al.). Microglial expression of lipoprotein lipase is required for supporting remyelination and repair through the clearance of lipid debris, a feature crucial for demyelinating disorders, including multiple sclerosis (Bruce et al.). An overview on the proteomic and metabolic profiling of vanishing white matter mouse astrocytes has been provided by Wisse et al.

The cell adhesion molecule with homology *to* L1 (CHL1) promotes cell proliferation, metastasis and migration in human glioma cells both *in vitro* and *in vivo* (Yang et al.). The origin of glioblastomas can be potentially associated wit adult neural stem cells (NSCs) and oligodendrocyte precursor cells (OPCs) serving as cells of origin for glioblastoma, as reviewed by Shao and Lui. In this aspect, another important finding demonstrates that ryanodine receptors (RyR3) mediate Ca^2+^ release from the endoplasmic reticulum in order to regulate OPC differentiation (Li et al.). Several other factors affect glial transformations. B-cell chronic lymphocytic leukemia (CLL)/lymphoma 11B f(Bcl11b) protein, a C2H2-type zinc finger transcriptional factor, negatively regulates glial progenitor cell differentiation through the repression of oligodendrocyte differentiation-associated genes (Wang et al.), whereas epithelial growth factor (EGF) enhances oligodendrocyte differentiation from glial progenitor cells (Yang et al.). Finally, Cloarec and colleagues show that administration of clodronate liposomes or maternal feeding with doxycyline, both result in alternation of microglial activity and abundance, improved survival in a rate model of cytomegalovirus infection (Cloarec et al.).

Glial cells were long thought to serve merely as the supporting cast and scenery behind the neuronal stars of the show. Relatively recent evidence, however, indicates that the glial cells are intimately involved in many of the brain's functions in health and disease. The intent of this Research Topic was to provide an update on recent developments in glial biology be that in health and disease.

## Author Contributions

All four authors contribute in writing the editorial and organizing the research topic.

### Conflict of Interest Statement

The authors declare that the research was conducted in the absence of any commercial or financial relationships that could be construed as a potential conflict of interest.

## References

[B1] Catenaccio A. Llavero Hurtado M. Diaz P. Lamont D. J. Wishart T. M. Court F. A. (2017). Molecular analysis of axonal-intrinsic and glial-associated co-regulation of axon degeneration. Cell Death Dis. 8:e3166. 10.1038/cddis.2017.489 29120410 PMC5775402

[B2] Kettenmann H. Hanisch U. K. Noda M. Verkhratsky A. (2011). Physiology of microglia. Physiol. Rev. 91, 461–553. 10.1152/physrev.00011.2010 21527731

[B3] Liddelow S. A. Guttenplan K. A. Clarke L. E. Bennett F. C. Bohlen C. J. Schirmer L.. (2017). Neurotoxic reactive astrocytes are induced by activated microglia. Nature 541, 481–487. 10.1038/nature21029 28099414 PMC5404890

[B4] Pekny M. Pekna M. (2014). Astrocyte reactivity and reactive astrogliosis: costs and benefits. Physiol. Rev. 94, 1077–1098. 10.1152/physrev.00041.2013 25287860

[B5] Pekny M. Pekna M. Messing A. Steinhäuser C. Lee J. M. Parpura V.. (2016). Astrocytes: a central element in neurological diseases. Acta Neuropathol. 131, 323–345. 10.1007/s00401-015-1513-1 26671410

[B6] Salter M. W. Stevens B. (2017). Microglia emerge as central players in brain disease. Nat. Med. 23, 1018–1027. 10.1038/nm.4397 28886007

[B7] Sofroniew M. V. (2014a). Astrogliosis. Cold Spring Harb Perspect Biol. 7:a020420. 10.1101/cshperspect.a020420 25380660 PMC4315924

[B8] Sofroniew M. V. (2014b). Multiple roles for astrocytes as effectors of cytokines and inflammatory mediators. Neuroscientist 20, 160–172. 10.1177/1073858413504466 24106265

[B9] Sun F. Lin C. L. McTigue D. Shan X. Tovar C. A. Bresnahan J. C.. (2010). Effects of axon degeneration on oligodendrocyte lineage cells: dorsal rhizotomy evokes a repair response while axon degeneration rostral to spinal contusion induces both repair and apoptosis. Glia 58, 1304–1319. 10.1002/glia.21009 20607865 PMC3045846

[B10] Verkhratsky A. Matteoli M. Parpura V. Mothet J. P. Zorec R. (2016). Astrocytes as secretory cells of the central nervous system: idiosyncrasies of vesicular secretion. EMBO J. 35, 239–257. 10.15252/embj.201592705 26758544 PMC4741299

[B11] Verkhratsky A. Nedergaard M. (2016). The homeostatic astroglia emerges from evolutionary specialization of neural cells. Philos. Trans. R Soc. Lond. B Biol. Sci. 371:20150428. 10.1098/rstb.2015.0428 27377722 PMC4938028

[B12] Verkhratsky A. Nedergaard M. (2018). Physiology of Astroglia. Physiol. Rev. 98, 239–389. 10.1152/physrev.00042.2016 29351512 PMC6050349

[B13] Verkhratsky A. Parpura V. (2016). Astrogliopathology in neurological, neurodevelopmental and psychiatric disorders. Neurobiol. Dis. 85, 254–261. 10.1016/j.nbd.2015.03.025 25843667 PMC4592688

[B14] Verkhratsky A. Rodríguez J. J. Parpura V. (2013). Astroglia in neurological diseases. Future Neurol. 8, 149–158. 10.2217/fnl.12.90 23658503 PMC3645493

